# Generating synthetic gait patterns based on benchmark datasets for controlling prosthetic legs

**DOI:** 10.1186/s12984-023-01232-6

**Published:** 2023-09-04

**Authors:** Minjae Kim, Levi J. Hargrove

**Affiliations:** 1 Department of Physical Medicine and Rehabilitation, Northwestern University, IL Chicago, USA; 2 Regenstein Center for Bionic Medicine, Shirley Ryan AbilityLab, IL Chicago, USA

**Keywords:** Generative adversarial network, Benchmark data, Impedance control, Synthetic impedance parameters

## Abstract

**Background:**

Prosthetic legs help individuals with an amputation regain locomotion. Recently, deep neural network (DNN)-based control methods, which take advantage of the end-to-end learning capability of the network, have been proposed. One prominent challenge for these learning-based approaches is obtaining data for the training, particularly for the training of a mid-level controller. In this study, we propose a method for generating synthetic gait patterns (vertical load and lower limb joint angles) using a generative adversarial network (GAN). This approach enables a mid-level controller to execute ambulation modes that are not included in the training datasets.

**Methods:**

The conditional GAN is trained on benchmark datasets that contain the gait data of individuals without amputation; synthetic gait patterns are generated from the user input. Further, a DNN-based controller for the generation of impedance parameters is trained using the synthetic gait pattern and the corresponding synthetic stiffness and damping coefficients.

**Results:**

The trained GAN generated synthetic gait patterns with a coefficient of determination of 0.97 and a structural similarity index of 0.94 relative to benchmark data that were not included in the training datasets. We trained a DNN-based controller using the GAN-generated synthetic gait patterns for level-ground walking, standing-to-sitting motion, and sitting-to-standing motion. Four individuals without amputation participated in bypass testing and demonstrated the ambulation modes. The model successfully generated control parameters for the knee and ankle based on thigh angle and vertical load.

**Conclusions:**

This study demonstrates that synthetic gait patterns can be used to train DNN models for impedance control. We believe a conditional GAN trained on benchmark datasets can provide reliable gait data for ambulation modes that are not included in its training datasets. Thus, designing gait data using a conditional GAN could facilitate the efficient and effective training of controllers for prosthetic legs.

**Supplementary Information:**

The online version contains supplementary material available at 10.1186/s12984-023-01232-6.

## Background

Prosthetic legs help people with amputations regain locomotion. A powered prosthetic leg is one of the most recently proposed types of prosthetic leg. It controls each amputated joint using an actuator that generates ambulation that is similar to that of individuals without amputation. The control of powered prosthetic legs is often achieved using a three-level hierarchical controller [1].

A high-level controller predicts human-intended ambulation modes, such as level-ground walking (LGW) and stair-climbing. On the other hand, a mid-level controller identifies the desired gait pattern and generates the control parameters for a low-level controller. A state machine [2], which is a well-known mid-level controller, usually extracts control parameters for an impedance controller. Lastly, a low-level controller controls motors with closed loops to satisfy the outputs (i.e., the control parameters) from a mid-level controller. For example, an impedance controller, which is a low-level controller, generates motor torques according to the impedance parameters generated by a mid-level controller, such as a finite state machine.

Recently, deep neural network (DNN)-based approaches have been proposed for control. The end-to-end learning capability of DNNs enables the precise and robust mapping of the input to the output parameters. Most DNN research is related to high-level controllers. For example, multiple ambulation modes and their transitions were recognized by convolutional neural networks (CNNs) using a depth sensor positioned on a powered hip exoskeleton [3]. Further, transfer learning [4] based on deep CNNs was used to enable intent prediction for multiple ambulation modes. A temporal CNN was used to extract the features of surface electromyography (EMG) signals to detect misclassification of the locomotion mode in lower-limb exoskeleton control [5]. Several mid-level controllers, such as reinforcement learning-based impedance controllers [6, 7], have been proposed. For instance, impedance parameters in a state machine were tuned to reproduce near-normal knee kinematics during walking [8]. In our previous study, a DNN model [9] was trained on the relationship between sensor data from a prosthetic leg and the output of a state machine-based impedance controller.

Obtaining and utilizing data for training is a significant challenge for these learning-based approaches. One possible approach is to obtain data from individuals with amputation or non-disabled participants using conventional controllers (i.e., bypass testing). In our previous work [9], We trained a DNN model to predict impedance parameters using state machine-controlled datasets obtained from transfemoral amputee users. This approach enabled seamless and intuitive transitions between various ambulation modes, including LGW, and ascending/descending stairs and ramps. However, the performance achieved with this approach may depend on the performance of the conventional controllers used. In addition, acquiring training data from users is time-consuming and onerous, particularly when targeting individuals with amputation.

Using benchmark datasets from individuals without amputation is another approach. Mathematical models can be trained on non-amputee datasets. In one study, an adaptive controller [10] based on the biomechanical analysis of non-disabled individuals enabled stair-climbing with variability in gait patterns, although noticeable differences were observed in the ankle joint angles between the sound side and the prosthetic legs. Further, models of the transition [11] from walking to stair-climbing and from stair-climbing to walking were designed using a publicly available lower-limb dataset [12] by interpolating untrained patterns using able-bodied kinematics models. A nominal gait pattern was used for the training of actor-critic-based reinforcement learning [13]. Adaptive parameter updating was used to significantly reduce gait tracking error and the difference in gait phase duration. Moreover, time-series data can be used to predict the movement of amputated joints (e.g., knee and ankle angles) from voluntary movements (e.g., thigh angle and vertical load on a prosthetic leg). In our previous work [14], a long short-term memory (LSTM)-based DNN model trained on a benchmark dataset that contained non-amputee ambulation data was used to extract impedance parameters for LGW at different step lengths. These studies demonstrate that gait patterns from non-amputee ambulation can be effectively used to train controllers for prosthetic legs.

In addition to the human gait being complicated and varied across individuals, differences between sensor characteristics [14], benchmark data, and prosthetic leg data must be compensated for. Furthermore, obtaining data is a challenge, particularly when more ambulation modes need to be considered. These data-driven models rely heavily on the availability and quality of data. Without sufficient data, the model may lead to inaccurate control.

In this study, we propose the generative adversarial network (GAN)-based generation of gait patterns to train DNNs for controlling prosthetic legs. The GAN, which is one of the most effective DNN applications, is widely used in image processing. Due to the versatility of GANs, they are also utilized to generate synthetic signals for various applications [15–17]. One advantage of GANs is their ability to generate output from a conditional input (i.e., a conditional GAN [22]). Hence, a GAN that is capable of generating a reliable gait pattern for ambulation modes that are not included in the training dataset could facilitate more efficient training of models for controlling prosthetic legs.

We trained a GAN using the gait pattern data, which included joint angles and vertical load (i.e., thigh, knee, and ankle angles, and weight on force plates) from benchmark datasets. Thereafter, the synthetic gait patterns for ambulation modes were generated, including those included and those not included in the training datasets. These patterns, which were derived from user inputs based on abstract knowledge, were extended to time-series data.

To demonstrate whether the GAN-based synthetic data could be used for control, we used the time-series data to train the DNN controller that was proposed in our previous work [14]. This DNN controller generates impedance parameters, including equilibrium angle, stiffness, and the damping coefficient for each joint, from vertical load and joint angles; the DNN controller trained on the benchmark LGW datasets successfully estimated the desired impedance parameters based on surface EMG data. However, in the current study, as surface EMG data were not available for the synthetic gait, synthetic time-series impedance parameters based on the literature were generated as the desired impedance parameters.

To demonstrate the control of prosthetic legs trained on synthetic data, we generated gait patterns for LGW, sitting-to-standing motion, and standing-to-sitting motion based on our abstract knowledge. Four individuals without amputation participated in the bypass testing (i.e., the participants wore a custom socket connected to a prosthetic leg) and successfully performed the intended ambulation modes.

The contributions of this study are as follows. First, we propose a GAN for gait pattern generation that is trained on publicly available benchmark datasets. Second, we demonstrate that the GAN is able to generate a reasonable gait pattern that is not included in the training datasets, and the generated pattern-based time-series data can be used to train DNN models for controlling prosthetic legs.

A GAN that is trained on benchmark datasets will provide reliable gait data for ambulation modes that are not present in the training datasets and that resemble the gait data of non-amputee individuals. Therefore, designing gait data using a GAN will facilitate the efficient and effective training of controllers for prosthetic legs by eliminating the need for training data from individuals.

## Methods

### Hardware and software

We used an open-source robotic leg (OSL) [18] to demonstrate the proposed control with the proposed method. The OSL includes a powered knee and an ankle that is actuated along the sagittal plane. In addition, the joint angles (thigh, knee, and ankle) and vertical load can be measured using self-contained sensors. We collected the sensor data and controlled the motors by providing impedance parameters every 5 and 25 ms, respectively.

The proposed networks for generating a synthetic gait pattern and controlling the OSL were trained using TensorFlow (v. 2.7.0, Google) in Python 3.8 on a laptop (Nitro 5, Acer) with Windows 11, an NVIDIA GeForce RTX 3050Ti Laptop GPU, and 32 GB DDR4 RAM. The training times for the GAN and the models that controlled the leg were 60 min and 40 min, respectively.

The DNNs for controlling the OSL were deployed on a Samsung Galaxy Z Flip 3 Android 12 smartphone. A Pyboard D-series SF6W was used as a bidirectional translation module between the OSL and the smartphone; the board converted commands from the OSL over CAN and transmitted them to the smartphone via USB serial communication, and vice versa. The execution time of the deployed DNN-based controller on the smartphone was approximately 9 ms. Thus, we ran two threads simultaneously: one functioned to parse the sensor data from the OSL and transmit the generated impedance parameters every 5 and 25 ms, respectively, and the other functioned to generate impedance parameters every 12 ms using the DNNs.

### Overall flow

**Fig. 1:**
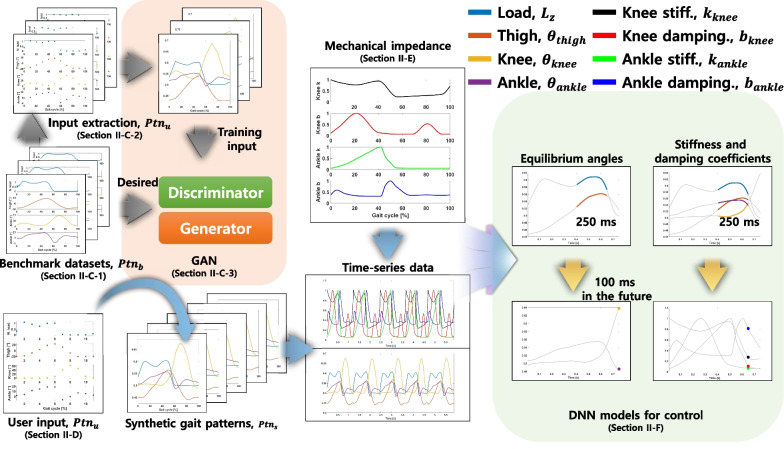
Overview of the proposed method. First, a GAN is trained using benchmark datasets. Second, a synthetic gait pattern is generated from user input using the GAN. Then, the set of gait patterns is converted into time-series data; the corresponding data for mechanical impedance parameters are generated. Finally, a DNN model that generates impedance parameters for controlling a prosthetic leg is trained on the generated time-series data

The proposed system (Fig. 1) comprises four parts. First, a GAN is trained using benchmark datasets that contain gait patterns (joint angles and vertical load) from non-amputee ambulation, denoted as $Ptn_{b}$. Second, a synthetic gait pattern ($Ptn_{s}$) is generated based on abstract knowledge ($Ptn_{u}$) using the generator, and the synthetic gait pattern is extended to time-series data. As the DNN-based controller requires not only time-series gait data, but also time-series impedance data, we generated time-series data for the mechanical impedance (i.e., stiffness and damping coefficient) of amputated joints (knee and ankle) based on the literature. Finally, DNN models for predicting impedance parameters (i.e., equilibrium angle, stiffness, and damping coefficient for each joint) are trained.

### GAN-based gait pattern

The purpose of the GAN is to generate continuous synthetic gait patterns ($Ptn_{s}$) of the vertical load and joint angles (i.e., the output of the generator) from our abstract knowledge, which is represented as discretized input data $Ptn_{u}$. The input data can be extracted from a continuous gait pattern or constructed manually. For example, in the case of LGW, we have the abstract knowledge that walking starts with thigh extension and that vertical load rapidly decreases to zero during the swing phase. We trained the GAN on benchmark datasets.

#### Benchmark datasets

Two benchmark datasets [12, 19] were used to train the GAN. These datasets contained time-series gait data from individuals without amputations. In these datasets, the joint angles (thigh, knee, and ankle) were collected using motion capture systems, and the vertical load was collected using force plates. Among the various ambulation modes in the benchmark datasets, we used only partial ambulation modes as we needed to transform the time-series data from the benchmark datasets to gait patterns for training the GAN: LGW and ascending/descending stairs from [19], and LGW and ascending/descending ramps from [12]. The other ambulation modes did not match our requirements. As an example of the data exclusion, although Reznick et al. [12] collected data for ascending stairs, only the joint angle data, without the force plate data, were included, so we did not consider their data.

Median gait patterns were obtained with a peak detection algorithm using the z-scores [20] for each data file, ambulation mode, or participant according to the format provided by the datasets. No additional processes, such as normalization or offset removal, were conducted. A total of 2145 and 100 gait cycles were obtained from [19] and [12], respectively. More details on this peak detection-based pattern extraction can be found in our previous work [21].

The continuous gait pattern (0% to 100%) was obtained every 0.5% gait cycle percentage. In conclusion, gait patterns ($Ptn_{b}$) of $R^{201\times 4}$ were obtained for the thigh, knee, and ankle angles and vertical load.

**Fig. 2:**
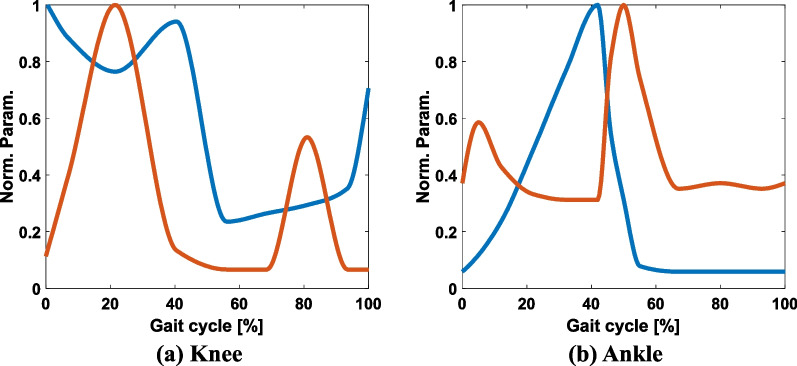
Stiffness (blue line) and damping coefficients (red line) for knee and ankle angles

**Fig. 3:**
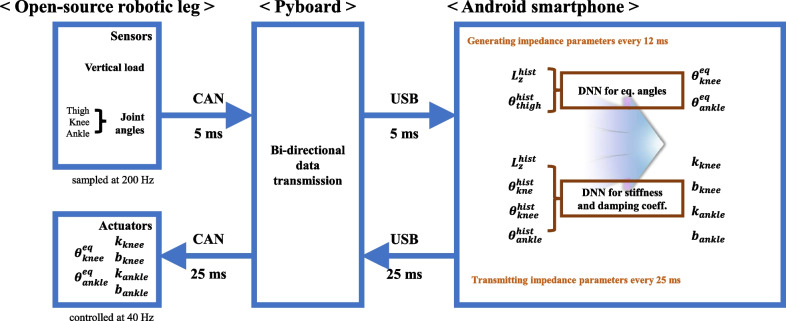
Proposed DNN-based control scheme including hardware. An Android smartphone collects sensor data every 5 ms and generates impedance parameters every 12 ms. The generated parameters are then transmitted to the prosthetic leg every 25 ms

**Fig. 4:**
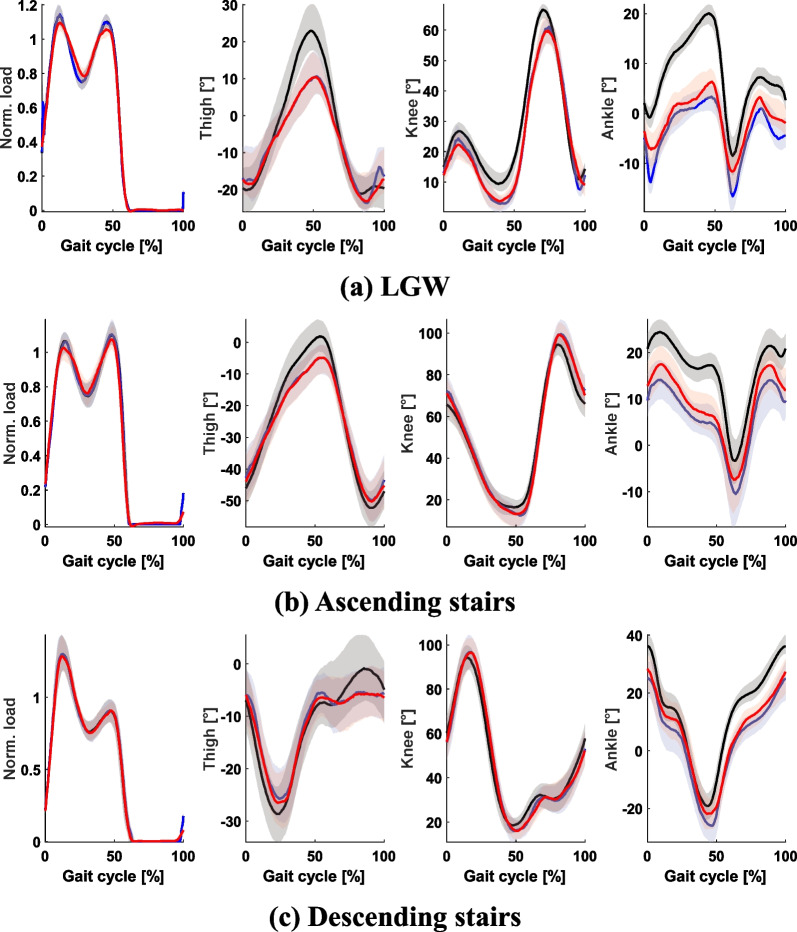
Patterns of the vertical load (first column) and joint angles (second to fourth columns). The black and blue lines indicate motion capture data and goniometer data, respectively, and the red line indicates a GAN-based synthetic gait pattern. The GAN generated gait patterns successfully, except for the ankle. All plots show 75th and 25th percentiles in lighter bands

**Fig. 5:**
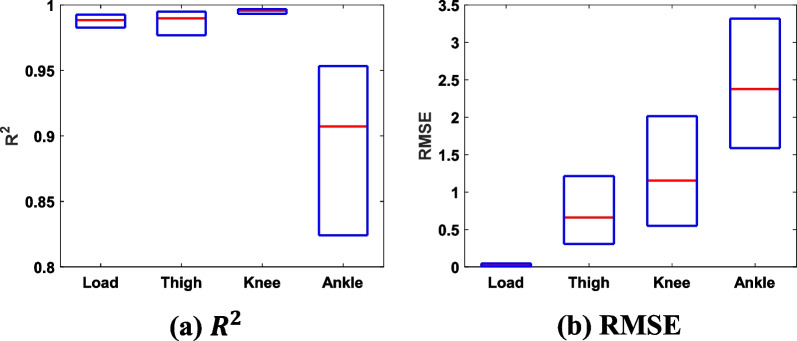
$R^2$ and RMSE between the goniometer data and corresponding synthetic gait patterns. For the vertical load, thigh, knee, and ankle, $R^2$ values were 0.99, 0.99, 0.99, and 0.91, respectively; RMSE values were 0.16, 0.66 $^{\circ }$, 1.15 $^{\circ }$, and 2.38 $^{\circ }$, respectively. A bar plot represents the 25th, 50th, and 75th percentiles

**Fig. 6:**
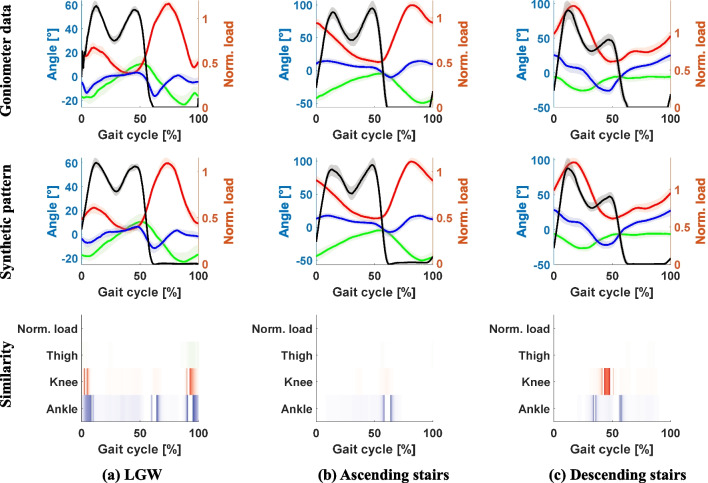
Structural similarity index between the goniometer data and the corresponding synthetic gait patterns. The first and second rows show the goniometer data and synthetic data, respectively. The vertical load, thigh, knee, and ankle angles are represented by black, green, red, and blue lines. The third row shows the dissimilarity between the data, with darker colors indicating lower similarity. Most of the differences were observed during phase transitions (e.g., from stance phase to swing phase). The overall similarities for LGW, ascending stairs, and descending stairs were 0.91, 0.97, and 0.96, respectively

**Fig. 7:**
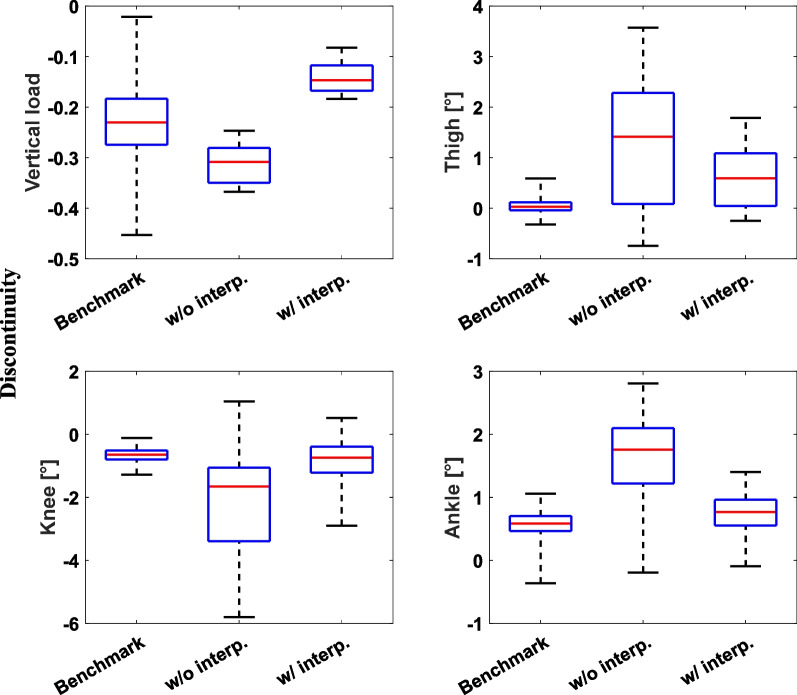
Discontinuity between the beginning and end of the gait cycle. The generated gait patterns, without interpolating sample points, showed a larger discontinuity compared to the benchmark datasets. However, implementing interpolations reduced the discontinuity to a level similar to that of the benchmark datasets. A bar plot represents the 25th, 50th, and 75th percentiles

#### Input and output configuration

Input-output pairs are needed to train the GAN. The output is the continuous gait pattern ($Ptn_{s}$) from 0% to 99.5% (i.e., $R^{200\times 4}$). During the training process, we extracted the discretized gait pattern ($Ptn_{u}$) every 10% from 0 to 100% (i.e., $R^{11\times 4}$) from the continuous gait pattern as input data.

As mentioned earlier, user input can also be represented as a discretized gait pattern that is manually designed. In other words, a user can provide any input in the form of a discretized gait pattern of $R^{11\times 4}$.

Irrespective of whether the input data of $R^{11\times 4}$ are extracted from a continuous gait pattern or obtained from user input, This data is linearly interpolated. The interpolation creates a continuous gait pattern obtained every 0.5% of the gait cycle percentage, from 0 to 99.5% (i.e., $R^{200\times 4}$). Thereafter, these interpolated data are used as input for the GAN.

#### Network architectures and parameters

The proposed GAN was adopted from Pix2Pix [22], although we modified its structure for our system. The size of the input and output to the GAN was $R^{200\times 4}$ (i.e., gait pattern from 0 to 99.5%). The reason for this dimension reduction (i.e., from 201 to 200) is that the input dimension should be a multiple of 2 because it is reduced by 1/8 of its original size during network processing. The input and output data were normalized for application to the GAN as follows:

1 $$\begin{aligned} v_n&= 0.1\frac{v}{g_v} + 0.5\nonumber \\ g_v&= \left\{ \begin{array}{ll} 36, &{} \text {if } v =\text {joint angle} \\ {\textit{Weight}}, &{} \text {if } v =\text {vertical load (benchmark)} \\ 48, &{} \,\,\text {if } v =\text {vertical load (bypass) } \end{array}\right. \end{aligned}$$

where *v* and $v_n$ denote the data and their normalized value, and $g_v$ denotes the gain. For example, when the knee angle is 108 $^{\circ }$, the normalized value is 0.8; when the thigh angle is − 72 $^{\circ }$, the normalized value is 0.3. In the case of the vertical load for bypass testing, to validate the robustness of the proposed method, we did not use divided load-cell data by body weight to obtain the normalized vertical load; rather, we simply divide the weight on the prosthetic leg by a constant value of 48. Therefore, the subject variability of the joint angles and the vertical load are maintained. For example, when the vertical load is 96 kg, the normalized value is 0.7.

The generator consists of:

Input-C16-C32-C64-T32-T16-T4-D200-Output

Here, Ck denotes a one-dimensional (1D) convolution layer with k filters, and Tk denotes a transposed 1D convolution layer with k filters. All convolution layers have the same padding, a kernel size of 2, a stride length of 2, and the LeakyReLU activation function. Dk denotes a dense layer of k units with sigmoid activation.

The discriminator consists of:

Input-C16-C32-C64-L80-Output

Here, Lk denotes an LSTM layer of k units with LeakyReLU activation.

The GAN was trained for 500 epochs ($\lambda$ [22] of 1000, with a batch size of 64, the ADAM optimizer with a learning rate of 0.01, and with the mean absolute error as the loss function).

### User input conversion into time-series data

The GAN converts user input, $Ptn_{u}$ ($R^{11\times 4}$), into an output gait pattern, $Ptn_{s}$ ($R^{200\times 4}$). By introducing variation into the user input, multiple output patterns can be obtained. We added the variation as follows:

2 $$\begin{aligned} Uv = U + 0.4 \times \sigma (U) \times r \end{aligned}$$

where *U* and *Uv* denote one variable (i.e., joint angle or vertical load) and its modified variable, respectively, $\sigma (U)$ denotes the standard deviation of the variable, and *r* denotes random values between − 1 and 1.

An output gait pattern ($Ptn_{s}$) is represented in terms of gait cycle percentage. This output of the GAN may contain noise, particularly when there are large differences between the user input and the data used for training. Therefore, we smoothed the output using cubic smoothing splines. The built-in MATLAB function *csaps* with a smoothing parameter of 0.1 was used for this purpose.

Subsequently, time-series data were obtained by connecting a set of gait patterns together. The transformed time-series data had a sampling frequency of 5 ms, which was the sampling frequency of the OSL used, and the time length of each gait pattern could be adjusted by providing a gait duration. For example, the original pattern from 0 to 99.5% ($R^{200}$) of each sensor data value could be converted into time-series data of 1.2 s comprising 240 sample points. To create smooth connections between the different gait patterns, one or two interpolating samples (i.e., 5 or 10 ms) were added between the connections. The number of interpolating samples (i.e., one or two) was randomly determined for every connection.

### Mechanical impedance from the literature

We adopted an impedance controller as the low-level controller because it provides robustness and safety in controlling the prosthetic leg. Accordingly, time-series data for the stiffness and damping coefficients were required to train the DNN models for controlling the leg. We adopted mechanical impedance from the literature [23, 24] for the ankle. In the literature, the authors estimated ankle joint impedance by employing system identification techniques across a gait cycle during LGW using wearable devices to collect information about joint kinematics. However, although a system for the knee was recently proposed [25], we could not find detailed impedance patterns for the knee. Therefore, we heuristically designed knee parameters based on the literature [26].

Based on the aforementioned studies, we designed the impedance patterns of the knee and ankle with a normalized value between 0 and 1, as shown in Fig. 2. Thereafter, we expanded the joint impedance in terms of the gait cycle percentage to the time-series data to match the gait pattern with the gait duration of the synthetic gait pattern.

### DNNs for control

An impedance controller was implanted to generate torques as follows:

3 $$\begin{aligned} \tau _i = -K_i(\theta _i - \theta ^{eq}_i) - b\dot{\theta }_i \end{aligned}$$

where $\tau _i$ denotes the joint torque; *i* denotes the knee or ankle joint angle ($\theta _i$); *k*, *b*, and $\theta ^{eq}$ denote the stiffness, damping coefficient, and equilibrium angle, respectively.

The DNN models for generating the impedance parameters were adopted from our previous work [14]. The control scheme for this approach is shown in Fig. 3. Two DNNs were trained independently: a DNN for predicting $\theta ^{eq}$ and a DNN for predicting *k* and *b*. Knee and ankle angles 100 ms in the future were considered as equilibrium angles. The equilibrium angles were predicted from the 250 ms history of the thigh angle and vertical load ($\theta ^{hist}_{thigh}$ and $L^{hist}_{z}$). The stiffness and damping coefficients of the knee and ankle were predicted from the 250 ms history of the joint angles ($\theta ^{hist}_{thigh}$, $\theta ^{hist}_{knee}$, and $\theta ^{hist}_{ankle}$) and vertical load ($L^{hist}_{z}$). Further, the output range of the stiffness and damping coefficients was 0 to 1. We scaled these outputs to the range of generic impedance parameters for the OSL, as presented in Table 1. In addition, we limited the output range of the equilibrium angles from 0$^{\circ }$ to 120$^{\circ }$ and from − 8$^{\circ }$ to 8$^{\circ }$ for the knee and ankle, respectively.

**Table 1:** Range of stiffness and damping coefficients

Joint	Impedance parameter	
	Stiffness (Nm/deg)	Damping coefficient (Nm s/deg)
Knee	1.5–5	0.05–0.5
Ankle	3–8	0.05–0.15

#### Network architectures and parameters

The network for predicting the equilibrium angles, which was trained using ADAM with a learning rate of 0.001, consists of:

Input-g0.02-c32-l20-d30-d10-d2-Output

Here, gk denotes the Gaussian noise layer with standard deviation k of the noise distribution, ck denotes a 1D convolution layer (k filters, same padding, kernel size of 8, stride length of 1, and sigmoid activation), lk denotes an LSTM layer of k units with sigmoid activation, and dk denote s dense layer of k units with sigmoid activation.

The network for predicting stiffness and damping coefficients, which was trained using RMSProp with a learning rate of 0.001, consists of:

Input-g0.02-c32-l20-d30-d10-d4-Output

These networks were trained for 15 epochs (with a batch size of 256 and the mean squared error as the loss function).

### Generating ambulation modes for control

We designed the input data ($Ptn_{u}$) manually based on our abstract knowledge of LGW, sitting-to-standing motion, and standing-to-sitting motion. Specifically, we adjusted the value of the data points of the discretized gait pattern every 10% from 0 to 100% using a custom MATLAB-based GUI application for all the joint angles and vertical load. The input data had to be provided in the format of a gait pattern. Thus, sitting-to-standing motion and standing-to-sitting motion were integrated (StS) and presented as one gait pattern.

For each ambulation mode (i.e., LGW and StS), we generated 10 datasets, each containing time-series data of patterns with 60 variations from a single input data value. The gait duration was randomly selected from 1 to 1.25 s and from 5 to 6.25 s for LGW and StS, respectively.

### Evaluation of GAN

We used a subset of the benchmark data [19] to evaluate the generated GAN. The dataset contained goniometer data as well as motion capture data (used to train the GAN) for the joint angles. Because of the differences in sensor characteristics, the goniometer and motion capture data show different joint angle values [21]. In conclusion, the subset containing goniometer data differs from the data included in the training dataset. The coefficient of determination ($R^2$) and root-mean-square error (RMSE) were calculated to evaluate the differences between the original goniometer data and the generated data.

Additionally, the structural similarity index [27] and the discontinuity between the original and generated patterns were evaluated.

Regarding the discontinuity, three different cases were examined. For the benchmark data, the difference was computed between the last and first sample data for the gait patterns ($R^{201\times 4}$). For the generated data without interpolation, the difference was measured between the last and first sample data of the gait patterns generated by the GAN ($R^{200\times 4}$). For the generated data with interpolation, the difference was calculated between the interpolated sample data and the first sample data of the GAN output patterns. Here, the interpolated data were derived from between the last and first sample data of the GAN output patterns.

### Experimental protocol

We demonstrated the proposed GAN-based control through real-time ambulation by non-amputee individuals (i.e., bypass testing). The purpose of the bypass testing was to demonstrate that the DNN-based controller, which was trained on synthetic gait patterns, was able to control the prosthetic legs as users intended. The data from the bypass users were not used for model training or tuning.

Four individuals (AB1 to AB4) participated in the testing, which was approved by the Northwestern University Institutional Review Board; all individuals provided written informed consent. AB1 and AB2 were brand-new participants, i.e., they had no experience with bypass testing. AB3 and AB4 had experience with bypass testing using conventional control methods, such as state machine-based [27] or phase variable-based [28] controllers. The tests were performed under the guidance and assistance of a licensed therapist and a certified prosthetist. Instruction and practice before data collection were minimized to confirm the validity of the proposed method. The brand-new participants (AB1 and AB2) walked using state machine-based controllers for approximately 5 min to familiarize themselves with the prosthetic leg.

The participants started by standing on the ground with the chair behind. After moving along 8 foot (2.4384 m) parallel bars, turning in place, and returning to the initial position, they performed the standing-to-sitting motion and the sitting-to-standing motion. The participants were allowed to grab the bar for safety while they executed the ambulation. Five repetitions of this activity were performed at a self-selected speed.

To evaluate the accuracy of the bypass testing, the smoothness of the joint angles (i.e., thigh, knee, and ankle angles) was evaluated in terms of the jerk for LGW and was compared to the benchmark data. The root mean square jerk in terms of the gait cycle was obtained using the following equation:

4 $$\begin{aligned} J = \sqrt{\frac{1}{2}\sum _{p=0}^{100\%}\theta _i(p)'''^2} \end{aligned}$$

where *p* denotes the gait cycle percentage, $\theta$ represent the joint angle, and *i* represents the thigh, knee, or ankle joint.

## Results

### GAN evaluation

The input data extracted from the benchmark goniometer dataset [19] were used to generate the gait patterns. The collected (i.e., original benchmark data) and generated gait patterns and the corresponding $R^2$ and RMSE are shown in Figs. 4 and 5, respectively. The ankle pattern showed a large difference compared with other sensor data. However, the results generally showed reasonable performance.

Figure 6 shows the gait patterns and their structural similarities. The overall similarities for LGW, ascending stairs, and descending stairs were 0.91, 0.97, and 0.96, respectively. As shown in the third row of the figure, dissimilarity was predominantly observed during the beginning, middle, and end of the gait cycles, where phase transitions typically occur. This result suggests that to improve gait generation, the input data for phase transitions should be carefully considered.

Figure 7 shows the discontinuity between the beginning and end of the gait cycle for the benchmark datasets and generated LGW gait patterns. The generated gait patterns exhibited higher discontinuity than the benchmark data; however, this discontinuity level decreased with the inclusion of additional interpolating points.

**Fig. 8:**
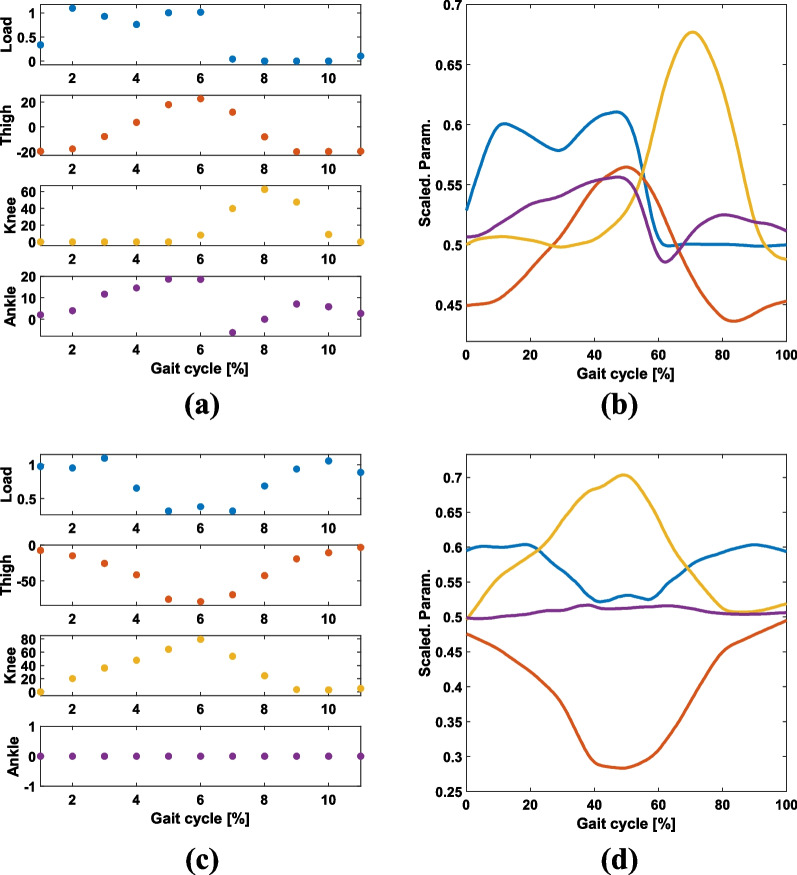
User inputs ($Ptn_{u}$) and corresponding synthetic gait pattern ($Ptn_{s}$) for LGW (**a**), (**b**) and StS (**c**), (**d**). Blue, red, yellow, and purple colors represent the weight, thigh angle, knee angle, and ankle angle, respectively. The GAN generated data for StS, although the mode was not included in the training dataset

We used the trained GAN to generate synthetic data for LGW and StS, as shown in Fig. 8. The GAN generated a reasonable pattern for StS, which was not included in the training dataset.

### Bypass testing

Four participants performed ambulation, including LGW and StS. They were able to perform all tasks without failure.

**Fig. 9:**
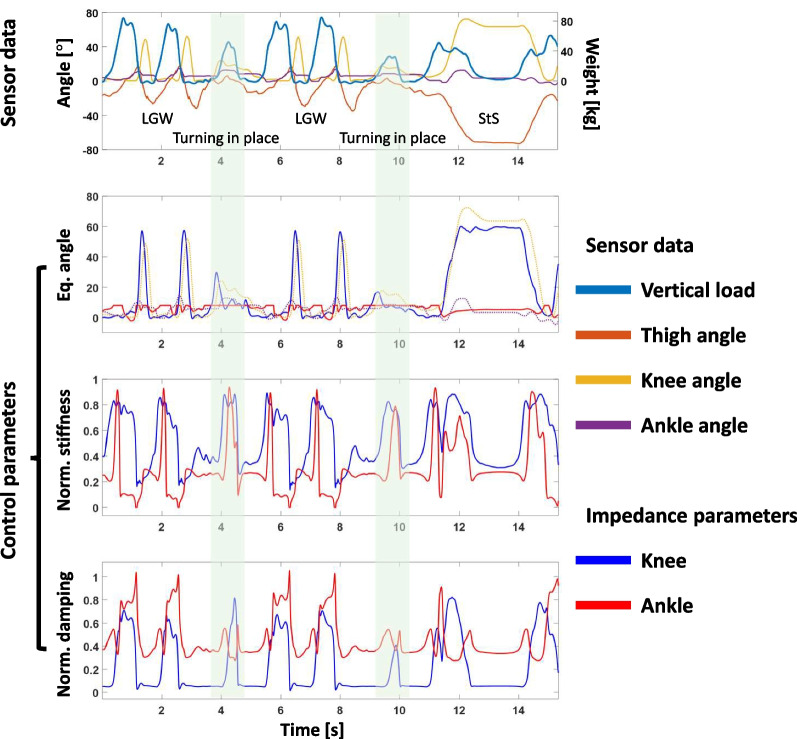
Example data from AB3. The participant was able to perform both LGW and StS

Figure 9 shows example data from AB3. The impedance parameters (stiffness, damping coefficient, and equilibrium angle) for each joint were generated from the pattern of the thigh angle and vertical load. Example data from other users can be found in Additional file 1.

**Fig. 10:**
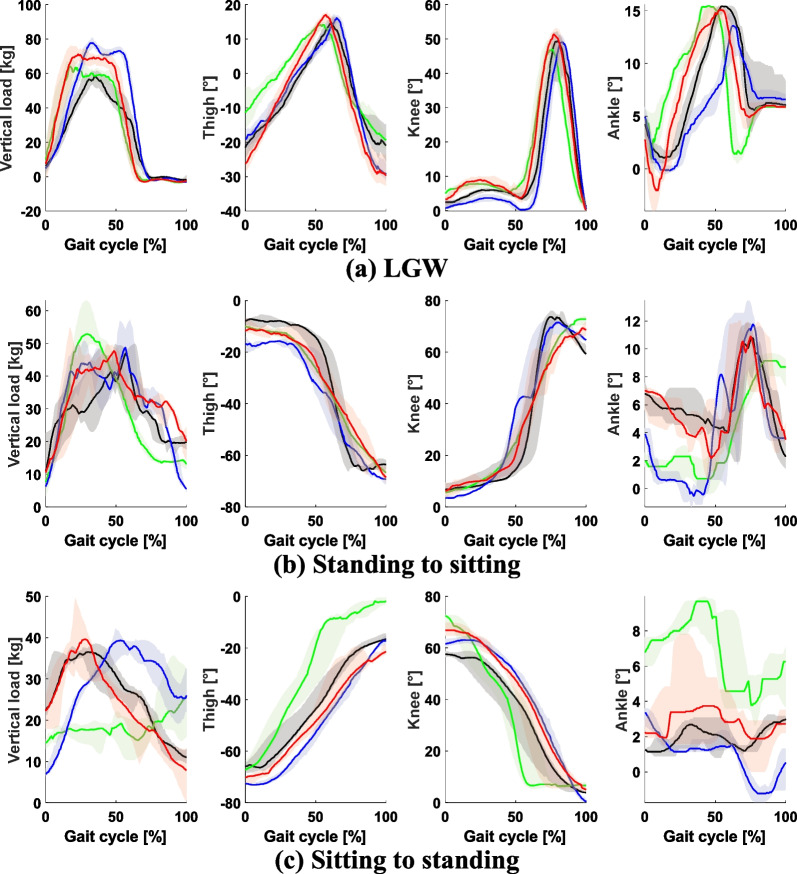
Gait pattern for LGW, standing-to-sitting motion, and sitting-to-standing motion. The black, green, blue, and red lines represent AB1, AB2, AB3, and AB4, respectively. The pattern of the knee and ankle varies according to the participants due to differences in the vertical load and thigh angles

**Fig. 11:**
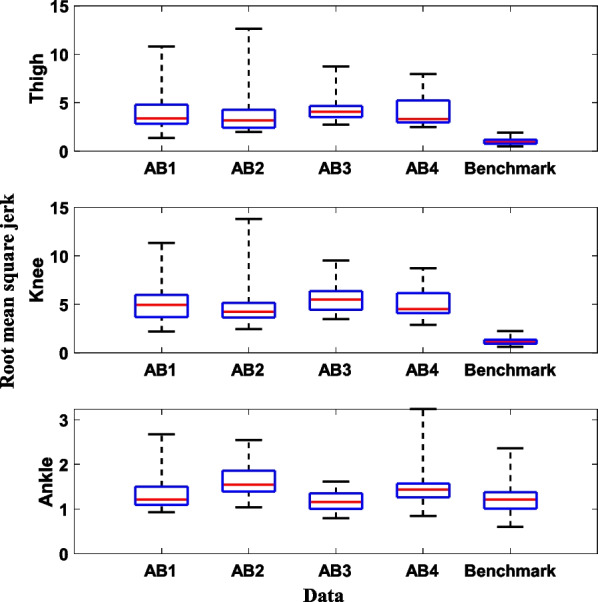
Smoothness (root mean square jerk) of LGW obtained during the bypass testing and data from benchmark datasets. The bypass users exhibited higher jerk than the able-bodied individuals (i.e., benchmark datasets) except the ankle angle. A bar plot represents the 25th, 50th, and 75th percentiles

As shown in Fig. 10, all participants have different patterns for vertical load and thigh angle, which are used as inputs to the DNNs for controlling the prosthetic leg. However, the gait smoothness of all four participants was worse than that of the benchmark dataset (Fig. 11) except the ankle angle. When comparing the median across all users, we found that the root mean square jerk for the thigh, knee, and ankle was 3.72, 4.34, and 1.15 times higher, respectively, than that of the benchmark datasets. In conclusion, although the users were able to perform the intended ambulation, their gait was not as smooth as that observed in the benchmark data.

## Discussion

The network architectures for controlling a prosthetic leg were adopted from our previous work [14]. However, we also added a 1D convolutional layer to improve the robustness of the model. Although the models with and without the convolution layer were not quantitively analyzed, the model with the convolution layer appeared to generate behaviors that were more stable. The DNNs were trained for 15 epochs, which was less than the 50 epochs in our previous work. Training and validation losses continuously decreased as the number of epochs increased. However, the trained model also showed less stable behavior when the epochs were increased. We speculate that overfitting to synthetic gait patterns degraded its performance because training data for the proposed model were not obtained from the prosthetic leg. This difference can potentially reduce stability and result in jerky movements. However, we believe that instability due to the difference between the prosthetic leg data and the synthetic gait patterns can be mitigated by utilizing better DNN architectures. For example, our DNNs include Gaussian noise layers to improve the stability of the system. As shown in Additional file 2: Video S2, the DNN-based controller without Gaussian noise layers shows unstable behaviors at the end of the swing phase.

We used the GAN to generate synthetic gait patterns and converted the patterns into time-series data to train the DNNs to control the OSL. However, this GAN-based pattern is not needed if a precise gait pattern is available. For example, generating continuous gait patterns from discrete input points using conventional curve-fitting or interpolation methods may be possible. Augmented patterns also can be obtained through various techniques, which include scaling or adding noise. As an example, our group has proposed an encoder-based data augmentation method [30]. However, these augmentation methods may not be able to generate data for a specific ambulation mode that is not included in the training datasets.

To demonstrate the capability of the GAN, we trained a GAN using an LGW-only dataset and then used it to generate synthetic gait patterns for ascending and descending stairs and ramps. We compared the GAN’s performance with other generation methods, including a general neural network and a machine learning-based regression method. The results can be found in Additional file 3. The patterns generated by our proposed GAN were closer to those in the benchmark dataset. However, we believe there may be better methods than the GAN, as many generative AI techniques have been proposed in recent years.

We used the discretized gait pattern as input data to the proposed GAN. The reason for discretization with low resolution is that we wanted to allow for user input by hand for ambulation modes that were not available in the training datasets. Although a higher resolution can lead to better performance, it would be relatively difficult to create high-resolution input data manually. We believe that input data for any ambulation mode can be manually generated from abstract knowledge. For example, in the case of ascending stairs, we generally know that a human hits the ground with a flexed knee, and the knee gradually extends in the stance phase. The thigh pattern is also similar to the knee pattern.

Connecting gait patterns to generate time-series data is a key factor in the proposed method because the DNNs for controlling legs were trained on time-series data. We added one or two interpolating sample points between the patterns, which enabled a smooth connection. The number of interpolating points was heuristically chosen, and we observed unstable behaviors when we increased the number of interpolating points. Adding an additional 10–50 interpolation points, for example, degraded performance, and extremely jerky motions were observed at the end of the swing phase.

We designed input data for the GAN to generate a pattern resembling that of non-amputee ambulation. However, there may be differences between non-amputee ambulation and the desired pattern for controlling prosthetic legs. Non-amputee ambulation data do not contain information about interactions between an individual and their surrounding environment. For example, although the ankle pushes the ground to provide push-off power in the late stance phase, it tends to be dorsiflexed because the ground is rigid and stiff. Therefore, the model trained on data with a dorsiflexed ankle in the late stance phase cannot provide adequate push-off power. These differences can cause discomfort to participants. In our testing, AB2 noted that the ankle was sometimes weak and thus did not provide sufficient push-off power in the late stance. AB2 also noted that standing-to-sitting motion felt like falling because the leg flexed earlier than intended.

Moreover, generating human-like patterns has the potential to reduce stability. For instance, in the case of LGW, small knee bending is typically observed during the mid-stance phase for non-disabled individuals (see Fig. 4). When this knee bending is reflected in a synthetic pattern, the prosthetic leg tends to execute sudden knee bending during the stance phase. Therefore, we designed the knee angle to be at zero degrees during the stance phase, as shown in Fig. 8.

A user inputting data to the GAN should have knowledge of the target systems as well as ambulation modes. Our input data were designed to generate a wide range of ankle movements for LGW (Fig. 4). However, the generated ankle movements were mostly dorsiflexed, as shown in Fig. 9. We speculate the reason for this result to be that the input data had a different thigh range from that of the OSL. The thigh angle dominantly affects the performance of the DNNs, as it is one of the two input data to the networks. Therefore, if input data are biased, the output will be biased as well. Hence, we speculate that there were differences in thigh angle between the output of the GAN (Fig. 4) and the actual behavior of the OSL (Fig. 10).

In conclusion, the desired pattern should be carefully considered for stable and reliable control. If the user input is generated manually, a designer should be confident about the pattern for the target ambulation modes and the expected system behaviors.

The initial structure of the GAN was adopted from Pix2Pix [22] for image-to-image translation. We modified the network architecture but retained the structure of the objective function in the original GAN. The hyperparameters of the network were heuristically chosen, and the performance of the GAN was significantly affected by changes in the hyperparameters. For example, we used LeakyReLU as the activation function, except in the last layer of the generator. The use of other activation functions degraded performance: the worst generation performance was obtained when the sigmoid was replaced with the LeakyReLU in the last layer of the generator. However, our model is not optimal; better architectures for signal generation may be possible. Instead of investigating the optimal network architecture, we focused on the robustness of the generator by adding post-smoothing.

For controlling a prosthetic leg, we trained two separate DNNs (one for equilibrium angles and the other for stiffness and damping coefficients), rather than one integrated DNN. One advantage of using separate networks is that we do not need to use the same data to train the impedance parameters. In particular, joint angles 100 ms in the future were considered as equilibrium angles. Therefore, we were able to extract the equilibrium angles from the time-series data for gait. In the case of the stiffness and damping coefficients, we adopted mechanical impedance from the literature because they could not be extracted from the sensor data. Additionally, the stiffness and damping coefficients were only available for LGW. In conclusion, had we used an integrated DNN for all impedance parameters, we would not have been able to train other ambulation modes, except for LGW.

In our previous work [14], stiffness and damping coefficients were generated from EMG data from lower limb muscles. During the preliminary testing, we asked individuals who were not participating in the experiments for this study which method felt more natural: the EMG-based or the mechanical impedance-based method. The individuals preferred the mechanical impedance-based method. However, neither method should be considered as ground truth. We believe that there will be a more efficient or optimal method for determining the stiffness and damping coefficients in the future. For example, the adoption of human-in-the-loop control [29] will provide subject-specific optimal control parameters for prosthetic legs.

As shown in Fig. 9, the model generated undesirable knee flexion during turning in place for all participants. We speculate that the model misinterpreted the turning-in-place mode as LGW because the mode was not included in the training dataset. Thus, DNNs for controlling prosthetic legs do not guarantee good performance for untrained ambulation modes. However, we demonstrated that GAN could be used to generate gait data for model training. Therefore, we believe that the performance of the DNN-based control can be improved by using synthetic gait patterns that represent various ambulation modes.

As shown in Fig. 10, the participants’ body weight was not fully on the prosthetic leg during StS. Participants were allowed to shift their weight on the parallel bars: thus, they tended to put weight on the parallel bars during StS, particularly during the sitting-to-standing motion. The intact leg that was connected to the socket of the prosthetic leg limited the range of motion for the prosthetic side; consequently, all participants noted that sitting with a prosthetic leg was difficult because the intact leg disturbed the motion.

The most significant limitation of this study is that stability has not yet been validated. Although the proposed method successfully generated gait patterns and controlled the prosthetic leg, the presented results could not guarantee whether the models possessed sufficient robustness for practical use; integrating with an impedance controller and limiting the ranges of the motor commands that were used in this study were insufficient. This issue should be resolved before testing on individuals with amputations is conducted.

In our future work, we will design various synthetic gait patterns and conduct demonstrations with individuals with transfemoral amputation. Our preliminary testing result is shown in Additional file 4: Video S4. In this testing, forward and backward walking were generated using the GANs. Here, input data were generated manually based on the patterns described in [30].

## Conclusion

In this study, we demonstrated that a GAN could be trained on publicly available benchmark datasets containing ambulation data for individuals without amputation to generate synthetic gait patterns based on abstract knowledge. Furthermore, ambulation modes that are not included in benchmark datasets can be generated. The gait pattern and mechanical impedance from the literature can be used to train DNN models for the impedance control of prosthetic legs. The proposed method was demonstrated with individuals without amputation; the participants were able to perform LGW and StS transitions. Future work will focus on providing a quantitative analysis of the proposed method and evaluating its performance for individuals with amputations.

### Supplementary information

**Additional file 1.** Example gait data from all participating users. Each user exhibits a different pattern for vertical load and angles, as well as for the corresponding impedance parameters.

**Additional file 2. Video S2.** This video demonstrates T-handle testing with and without Gaussian noise layers in the DNN-based controller. The prosthetic leg exhibits unstable behaviors without the Gaussian noise layers.

**Additional file 3.** This document presents a performance comparison in terms of generating gait patterns for ambulation modes that were not included in the training dataset. The proposed GAN demonstrates better generation than a general DNN method and a machine learning-based regression.

**Additional file 4. Video S4.** This video demonstrates the control of a prosthetic leg using the proposed method. The first part is the bypass testing; in this testing, we used GAN-based synthetic data to train a DNN-based controller for LGW, standing-to-sitting motion, and sitting-to-standing motion. The second part is our preliminary testing in individuals with transfemoral amputation. In this testing, a controller was trained on a synthetic gait data for walking forward and backward.

## Data Availability

All data generated or analyzed during this study are included within the article.

## Abbreviations

CNN: Convolutional neural network
DNN: Deep neural network
GAN: Generative adversarial network
LGW: Level-ground walking
LSTM: Long short-term memory
OSL: Open-source robotic leg
RMSE: Root-mean-square error
$R^2$: Coefficient of determination
StS: Sit-to-stand and stand-to-sit
1D: One-dimensional
