# Supplementary figures and images for: Generating synthetic gait patterns based on benchmark datasets for controlling prosthetic legs
Source: J Neuroeng Rehabil. 2023 Sep 4;20:115. doi: 10.1186/s12984-023-01232-6 (PMC10476332; doi:10.1186/s12984-023-01232-6)

AB1

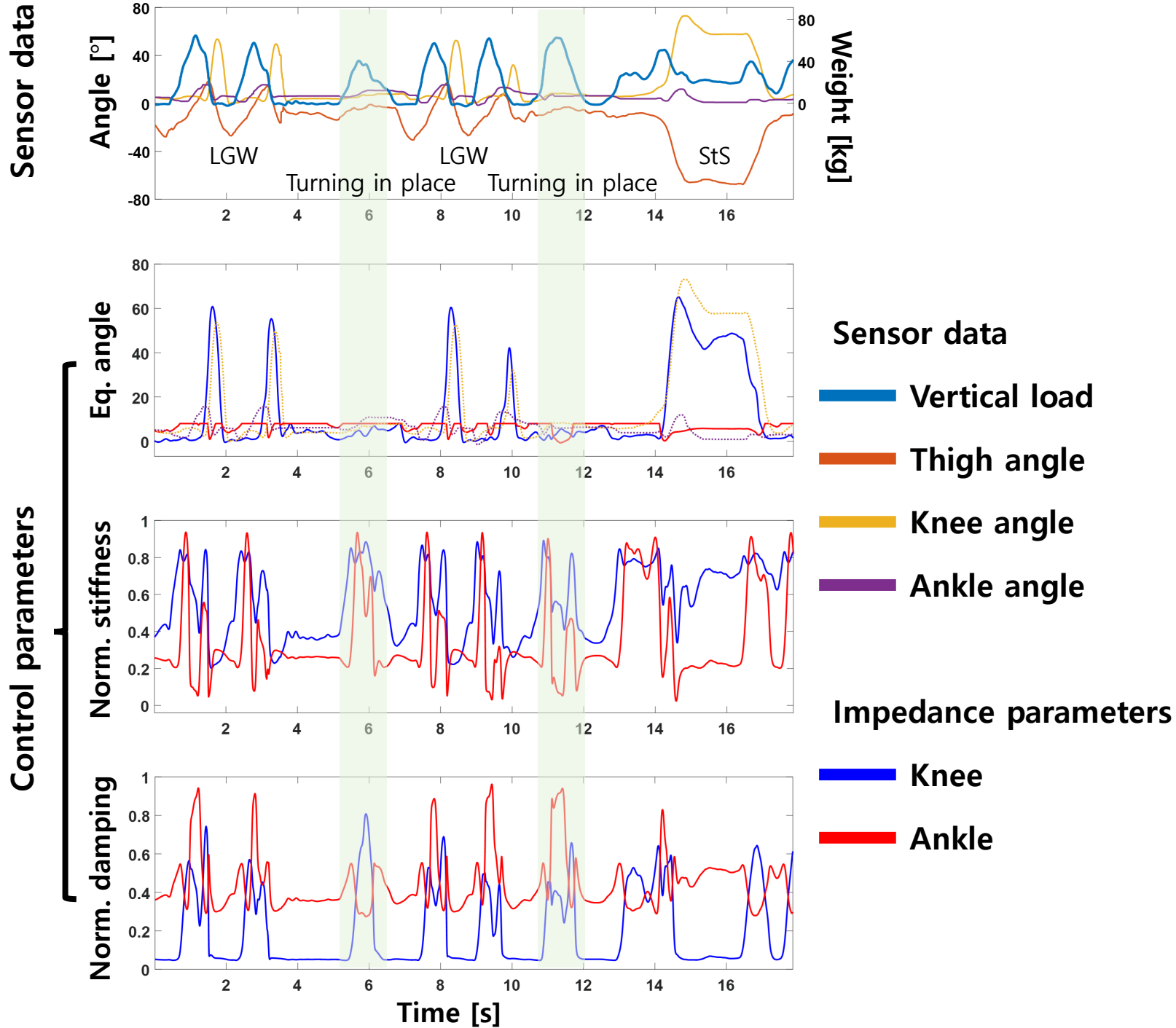

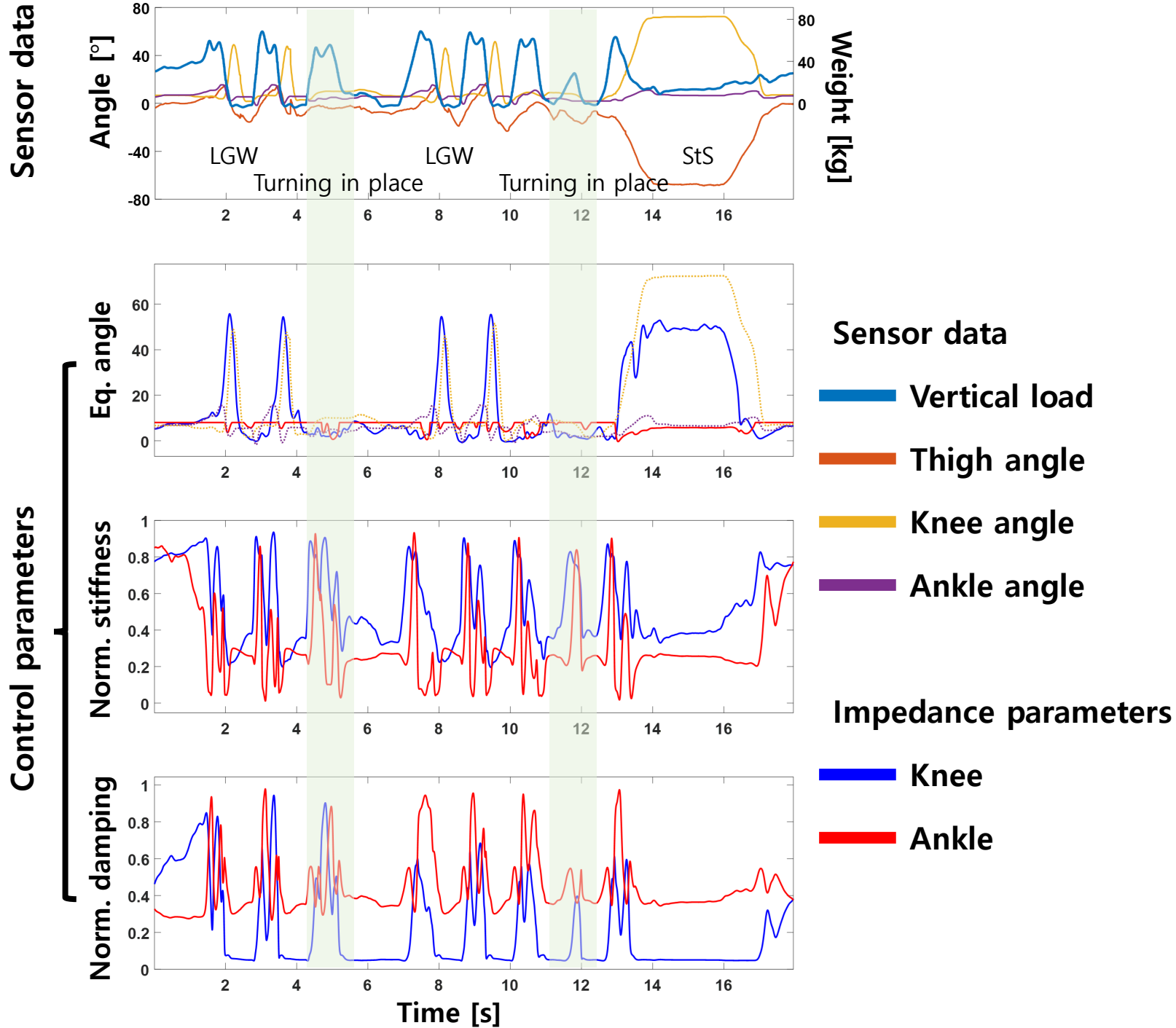

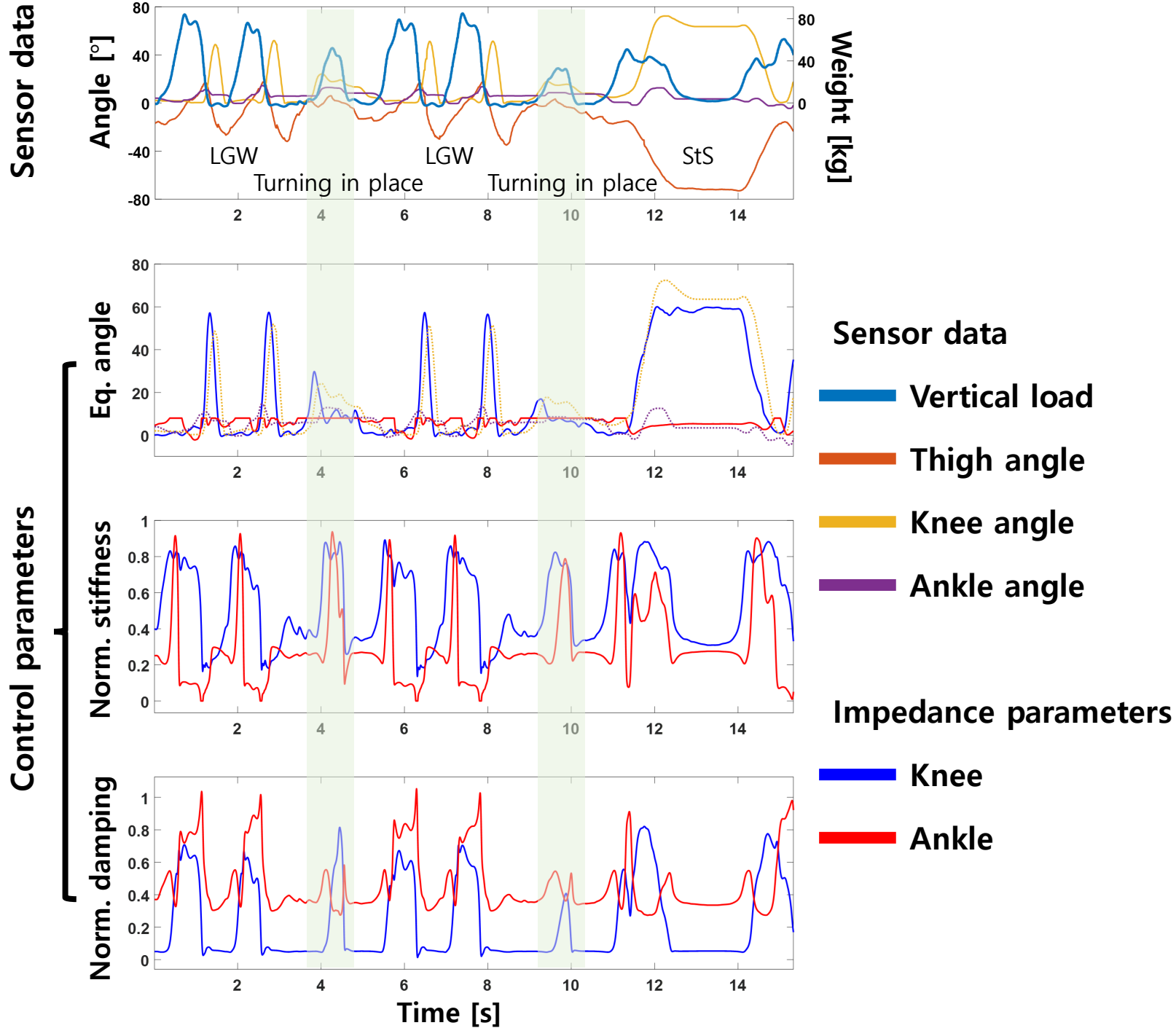

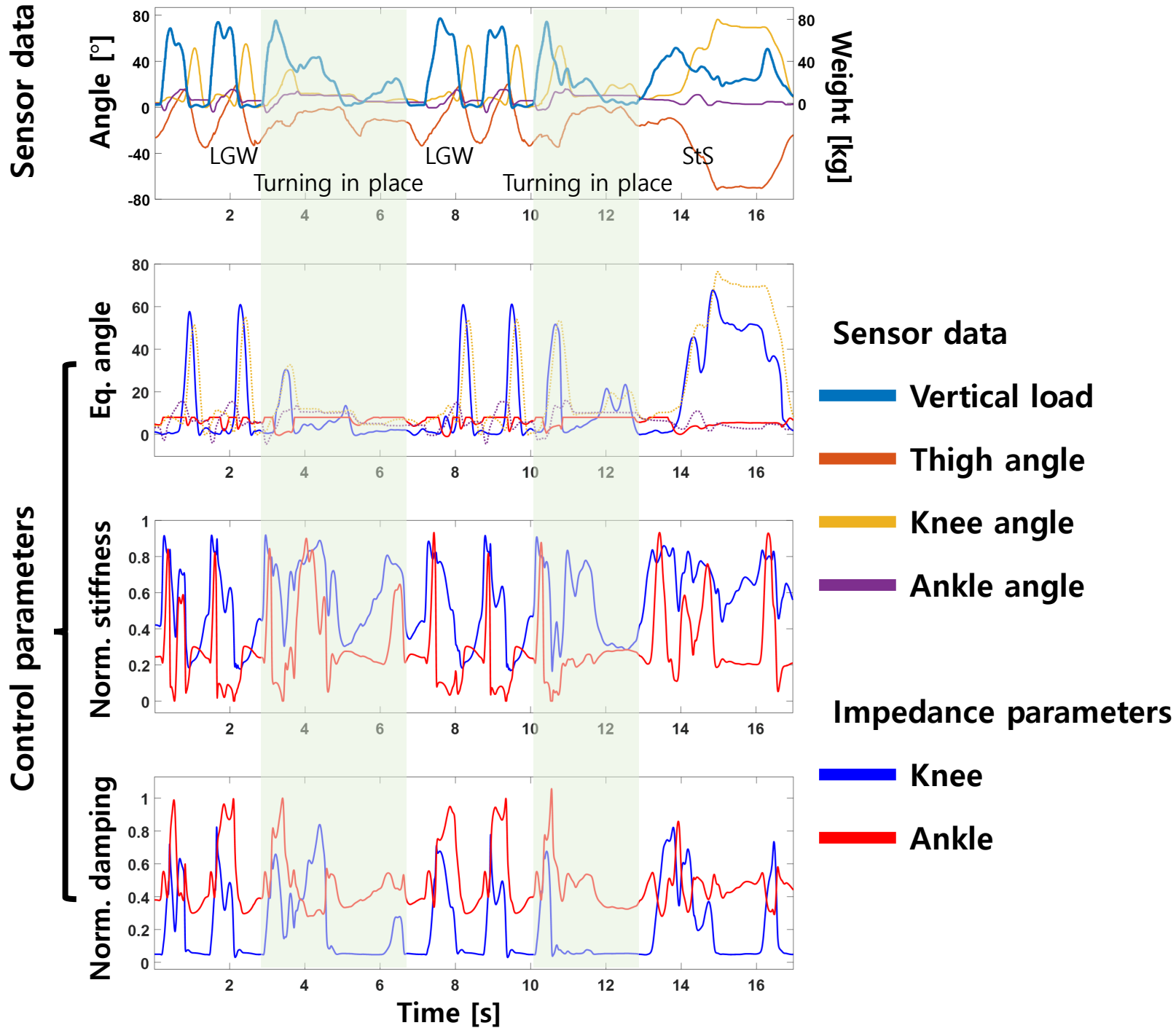

Supplement: Supplementary file 1 — Additional file 1. Example gait data from all participating users. Each user exhibits a different pattern for vertical load and angles, as well as for the corresponding impedance parameters. [file 12984_2023_1232_MOESM1_ESM.pdf]
