# Supplementary material for: Generating synthetic gait patterns based on benchmark datasets for controlling prosthetic legs
Source: J Neuroeng Rehabil. 2023 Sep 4;20:115. doi: 10.1186/s12984-023-01232-6 (PMC10476332; doi:10.1186/s12984-023-01232-6)
Supplement: Supplementary file 3 — Additional file 3. This document presents a performance comparison in terms of generating gait patterns for ambulation modes that were not included in the training dataset. The proposed GAN demonstrates better generation than a general DNN method and a machine learning-based regression. [file 12984_2023_1232_MOESM3_ESM.pdf]

To demonstrate the capability of generating synthetic gait patterns not included in the training datasets, we tested three approaches: the proposed *GAN*, a general deep neural network (*Fit*), and polynomial regression (*Rgr*). We extracted five sub-datasets from benchmark datasets: level-ground walking (LGW), ascending ramps (upramps), descending ramps (downramps), ascending stairs (upstairs), and descending stairs (downstairs). Figure 1 shows scheme of model training and extraction of synthetic patterns (Ptn_s)

Fit employs the same network architecture as the generator of the *GAN*, and it learns the relationship between Ptn_b and Ptn_u using only the generator, as is typical in deep learning. *Rgr* was trained using Scikit-learn PolynomialFeatures with a maximum degree of 5 and default values for all other parameters.

We trained *GAN* and *Fit* on the LGW datasets, while *Rgr* was trained on each sensor data (thigh angle, knee angle, ankle angle, or vertical load) for each ambulation mode. For example, to obtain thigh angle in Ptn_s of upstairs, only thigh angle in Ptn_u of upstairs was used to train the *Rgr*. As a result, this produced a total of 20 *Rgr* models.

Figure 2 shows comparisons between Ptn_b and Ptn_s generated using the three approaches (*GAN*, *Fit*, and *Rgr*); Figure 3 and Figure 4 show their correlation and structural similarity index, respectively. The black, red, blue, and green lines represent the benchmark data, *GAN*, *Fit*, and *Rgr*, respectively.

GAN demonstrated superior performance in generating synthetic patterns (Ptn_s) that closely resembled to the benchmark data, Ptn_b. However, the performance of generation can be affected by the network architecture and parameters, so we cannot claim that the proposed method is the optimal approach. Nevertheless, we believe that the use of generative networks, not limited to the GAN, can generate reliable patterns that are not present in the training datasets.

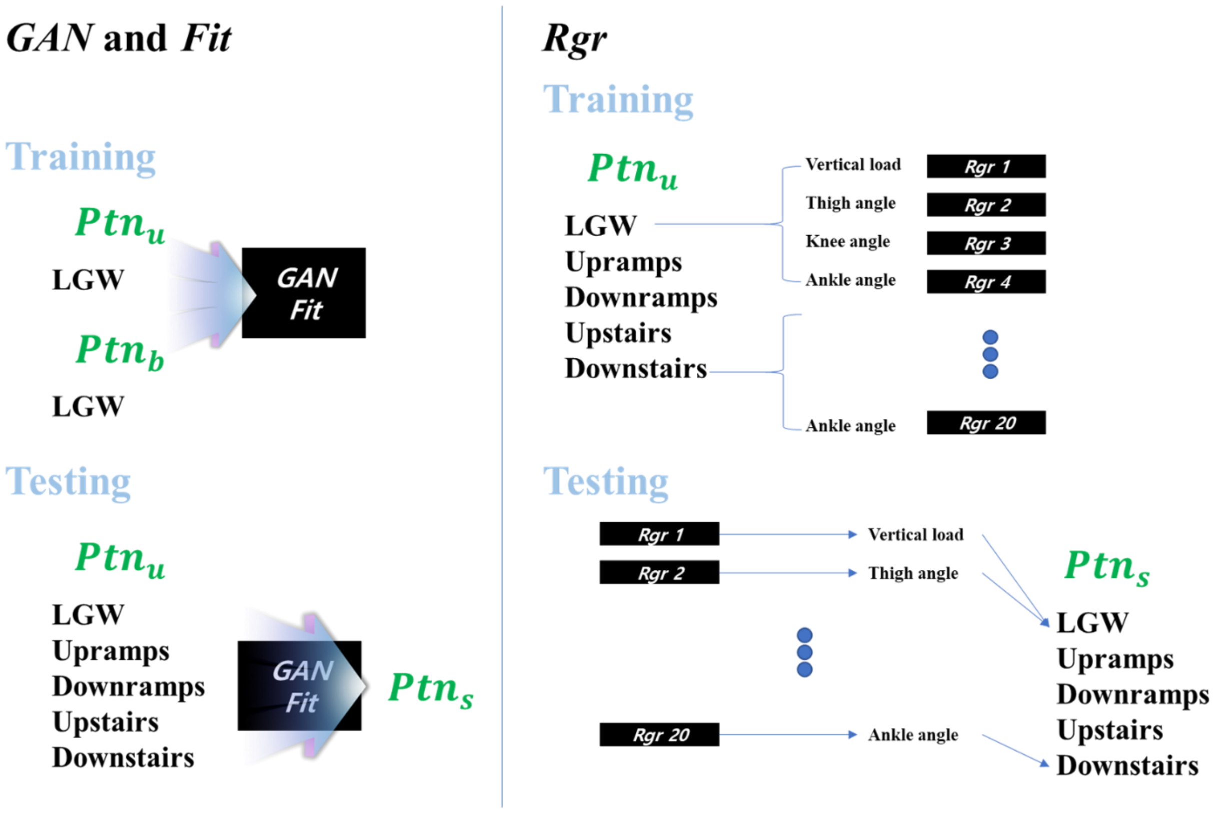

Figure 1. Model training and synthetic gait data extraction of each approach.

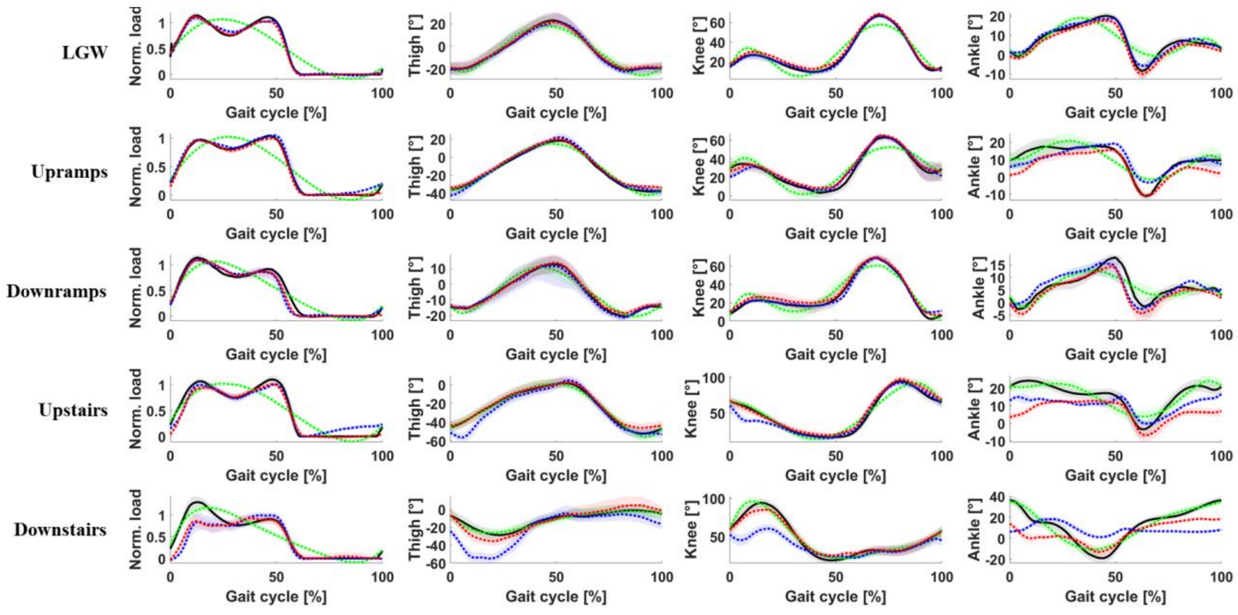

Figure 2. Gait patterns of benchmark datasets and corresponding synthetic data using GAN (red), Fit(blue), and Rgr (green)

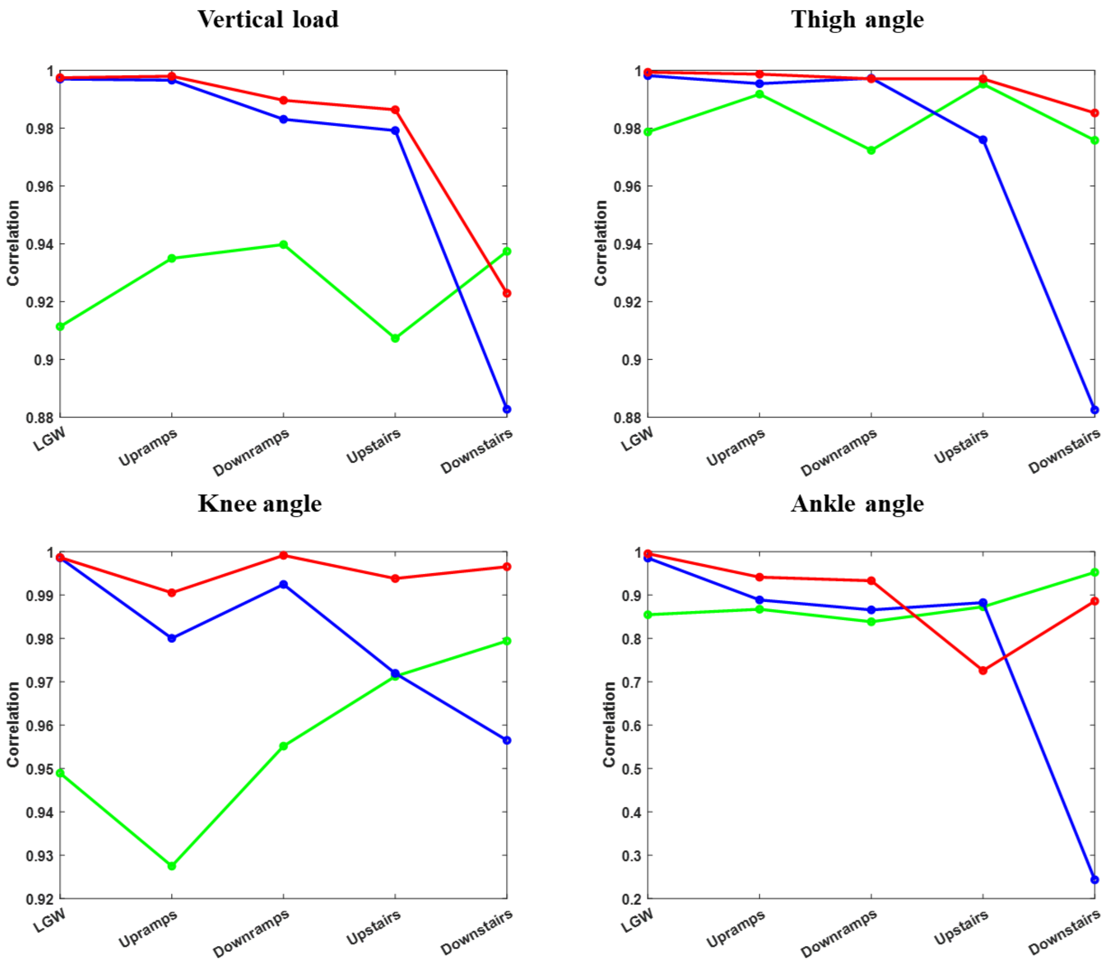

Figure 3. Correlation between benchmark data and synthetic gait data of each sensor.
The red, blue, and green lines represent GAN, Fit, and Rgr, respectively.

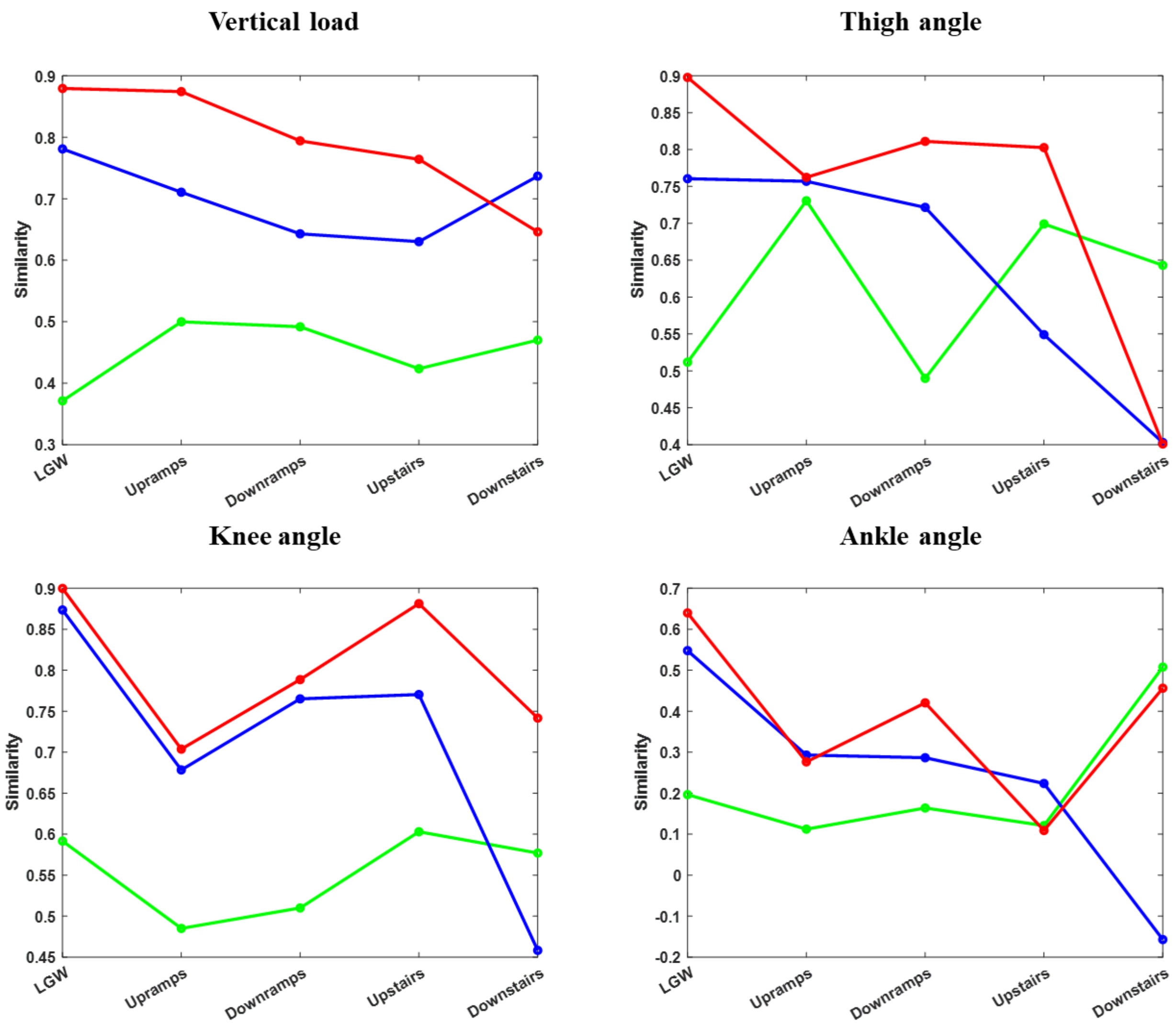

Figure 4. Structural similarity index between benchmark data and synthetic gait data of each sensor. The red, blue, and green lines represent GAN, Fit, and Rgr, respectively.
